# Exploring significant gender disparities in esophageal cancer risk: Insights from Mendelian randomization analysis and global burden of disease data

**DOI:** 10.1097/MD.0000000000047800

**Published:** 2026-02-28

**Authors:** Jiale Cui, Rong Zhang, Xiaomeng Zhang, Ziyi Zhang, Meixuan Li, Rui He, Peirui Jiang, Lei Li

**Affiliations:** a School of Basic Medicine, Shanxi Medical University, Tai Yuan City, Shan Xi Province, China; b The Gynecology and Obstetrics Department, Shanxi Provincial People’s Hospital, City Tai Yuan City, Shan Xi Province, China; c School of Nursing, Shanxi Medical University, Tai Yuan City, Shan Xi Province, China; d School of Public Health, Shanxi Medical University, Tai Yuan City, Shan Xi Province, China; e The Radiotherapy Department, Shanxi Provincial People’s Hospital, Tai Yuan City, Shan Xi Province, China.

**Keywords:** esophageal cancer, gender difference, global burden of disease, Mendelian randomization

## Abstract

Esophageal cancer (EC) is responsible for approximately 508,000 deaths worldwide each year. Emerging evidence suggests significant gender disparities in EC-related mortality. This study aims to explore the underlying causes of these differences. In this study, global mortality rates of EC were obtained from the global burden of disease database. Several factors strongly associated with EC risk were identified and treated as exposures. Mendelian randomization analysis was then conducted to evaluate the potential causal relationships between EC risk and testosterone levels (TLs), body mass index, smoking, alcohol consumption, red meat intake, and vegetable consumption. The results showed that higher TLs (*P* = .03; beta = 0.07; 95% confidence interval, 0.006–0.13) and smoking (*P* = .03; beta = 0.39; 95% confidence interval, 0.02–0.78) were significantly and positively associated with an increased risk of EC. No causal associations were observed between EC risk and the other investigated factors. Survival analysis revealed that PTEN and PIK3CA proteins had no statistically significant impact on the survival of EC patients (PTEN: Log-rank *P* = .89, HR = 1.1; PIK3CA: Log-rank *P* = .97, HR = 0.99). Meanwhile, TP53 protein suggested a potential adverse effect on survival (Log-rank *P* = .057, HR = 2.5). Significant gender disparities in the risk of EC may be attributed to the direct causal effects of smoking and elevated TLs in males. In contrast, factors such as alcohol consumption, body mass index, and dietary habits do not appear to have a significant impact on EC risk. It was also observed that the causal relationship between TLs and the risk of EC may be mediated by the TP53 protein.

## 1. Introduction

Esophageal cancer (EC) is the sixth leading cause of cancer-related mortality worldwide, characterized by high morbidity and poor prognosis. It affects over 450,000 individuals annually and poses a significant global public health challenge.^[1,2]^ EC-related mortality shows a marked gender disparity, with incidence rates approximately 2 to 3 times higher in men than in women, and it is more prevalent among the elderly population.^[3]^ Lifestyle factors such as smoking, alcohol consumption, and dietary habits have been implicated in the development of EC.^[4]^ Previous studies have shown that current smokers are at a significantly higher risk of developing EC compared to nonsmokers.^[5]^ Moreover, average weekly alcohol consumption exceeding 170 grams has been associated with a substantially increased risk of EC.^[6]^ Epidemiological evidence also suggests an inverse relationship between vegetable intake and EC risk, supporting dietary recommendations that promote increased vegetable consumption and reduced red meat intake for individuals at elevated risk.^[7]^ However, the causal relationships between these risk factors and EC development remain to be conclusively established.

This study utilized data from the global burden of disease (GBD) database to estimate the global mortality burden of EC and to preliminarily identify factors strongly correlated with EC risk. To assess potential causal relationships, Mendelian randomization (MR) analysis was conducted, focusing on gender differences, smoking, alcohol consumption, red meat intake (including pork and beef), and vegetable consumption. Simultaneously, we performed survival analysis on 3 proteins (PTEN, PIK3CA, TP53) that are demonstrably associated with testosterone levels (TLs),^[8–10]^ aiming to tentatively hypothesize the molecular mechanisms underlying the causal relationship between TLs and the risk of EC.

MR is an analytical method that uses genetic variants as instrumental variables (IVs) to evaluate the causal effects of modifiable exposures on health outcomes.^[11]^ Because genetic variants are randomly allocated at conception, MR analyses can reduce bias from confounding and reverse causation, offering more reliable causal inferences than traditional observational studies.^[12]^ In this study, a 2-sample MR analysis was conducted to investigate the potential causal relationships between EC risk and various modifiable risk factors.

## 2. Materials and methods

### 2.1. Retrieval of GBD data

In this ecological time-series study, EC-related data were obtained from the 2021 GBD study. The dataset includes information on EC prevalence, mortality, disability-adjusted life years (DALYs), age-standardized prevalence rates, age-standardized mortality rates (ASMRs), DALY rates, and corresponding 95% uncertainty intervals from 1990 to 2021. In addition, data on the global number of deaths, mortality rates, and DALYs attributable to age- and sex-specific risk factors were retrieved and analyzed.^[13–15]^

### 2.2. Genetic variants associated with different exposure factors

All the GWAS data used for the univariate MR analysis and meta-analysis of the exposure of interest were retrieved from the IEU database (https://gwas.mrcieu.ac.uk/). The following datasets associated with the different exposure factors were retrieved in this study: alcohol consumption: ieu-a-1283, ukb-a-31, ukb-b-5779, and ukb-b-16878; body mass index (BMI): ieu-a-94, ieu-a-835, ieu-a-974, ieu-b-40, ieu-b-4815, ieu-b-4816, ieu-a-248, ukb-b-2303, and ukb-b-19953; meat intake: ukb-b-2862, ukb-b-5640, and ukb-b-6324; consumption of vegetables: ukb-b-1996 and ukb-b-8089; smoking: ukb-a-16, ukb-b-17, ukb-a-225, ukb-a-223, ukb-a-2134, and ukb-b-6244; and TLs: ieu-b-4864, ieu-b-4869, and ukb-d-30850.

IVs were selected from among all single nucleotide polymorphisms (SNPs) showing correlation with the exposures, meeting the GWAS statistical significance threshold (*P* <1 × 10^−8^). To ensure the independence of IVs, a linkage disequilibrium analysis was performed with a threshold of *r*^2^ <0.001. Furthermore, it was confirmed that each IV had an F-statistic value exceeding 10 (*F* >10), thereby affirming the validity of the IVs.

### 2.3. GWAS summary data for EC

The dataset pertaining to EC was retrieved from a scholarly article published in *Nature Genetics* in 2021, which reported the results of primary analysis by Sakaue et al.^[16]^ This comprehensive dataset contains data for 476,306 samples and 24.2 million SNPs.

### 2.4. Statistical analyses of GBD data

The global burden of EC was evaluated in this study based on the ASMRs and age-standardized DALY rates. In addition, the mortality and DALY data were comprehensively documented in this study. The age-standardized rates (per 100,000 individuals) across all age groups, the specific mortality rates for defined age groups, and the corresponding numerical data were extracted from the 2021 GBD database. These data are presented as estimated values, complete with the 95% uncertainty intervals.^[14,15]^ The ASR was calculated using the following formula: $ASR=\frac{\overset{N}{\underset{i=1}{\sum }}a_{i}w_{i}}{\overset{N}{\underset{i=1}{\sum }}w_{i}}$. Where a_i_ represents the age-specific rate in the i^th^ age group, w_i_ denotes the number of individuals in the standard population within each age group, and the variable *N* signifies the total number of age groups.

The estimated annual percentage change (EAPC) in the ASR was computed to quantify the trend in the fluctuations in EC from 1990 to 2021. A linear regression model, expressed as y = α + βx + ε, was used for the calculations, where y represents the natural logarithm of the ASR, x denotes the calendar year, and ε is the independent, normally distributed error term. The EAPC was subsequently determined using the formula: EAPC = 100 × (e^β^–1). The EAPC, in conjunction with its 95% confidence interval (CI), was utilized to delineate the trend within the specified time frame [τ_j—1_, τ]. A statistically significant decline was indicated over the observation period when the upper bound of the EAPC (95% CI) was below zero. Conversely, a statistically significant upward trend was suggested if the lower bound of the EAPC (95% CI) exceeded zero. However, the change in the ASR was regarded as statistically insignificant when the EAPC (95% CI) encompassed zero, and implied that the observed trend did not differ statistically from no change.

### 2.5. Statistical analyses of MR

**Figure 1.**
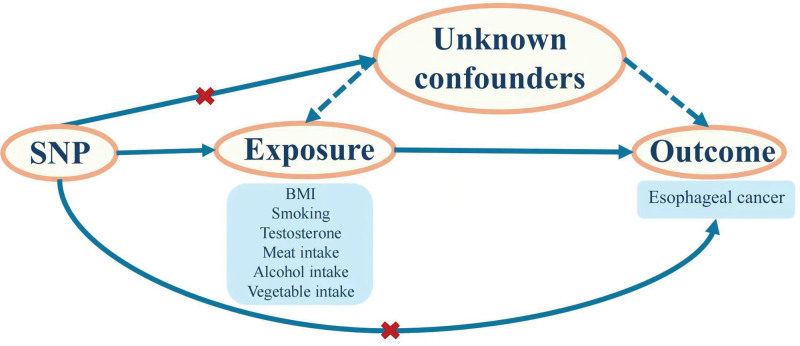
The assumptions of MR analysis: (i) genetic variant is associated with the exposure in question (correlation assumption). (ii) The genetic variant influences the outcome solely through its effect on the exposure, without having any direct effects on the outcome (exclusion restriction assumption). (iii) The genetic variant in question is not associated with other confounding factors that influence the outcome (independence assumption). MR = Mendelian randomization.

In this study, we employed a 2-sample MR approach to investigate the causal relationship between risk factors and the incidence of EC. MR research genetic variants as IVs, and several criteria must be fulfilled to validate the selection of these variants as IVs. The key criteria include:

1. The genetic variant in question demonstrates a significant association with the exposure under investigation (correlation assumption).
2. The genetic variant exerts its influence on the outcome exclusively via its impact on the exposure, devoid of any direct influence on the outcome itself (exclusion restriction assumption).
3. The genetic variant under consideration does not exhibit an association with other confounding variables that affect the outcome (independence assumption)^[17]^ (Fig. 1).

The MR analyses undertaken in the present investigation predominantly relied on the IVW method.^[18,19]^ To ensure the robustness of the causal inferences, we further employed the WM approach and the MR-Egger regression analysis. The IVW approach treats each SNP as a distinct natural experiment affecting the outcome, amalgamating these effects to estimate the overall causal relationship. The fixed-effect IVW model is designed to provide an unbiased estimation in the absence of horizontal pleiotropy or when such pleiotropy is balanced.^[20]^ Statistical significance was determined by a *P*-value <.05 from the IVW model, which was also consistent with the direction of effect indicated by the MR-Egger regression and WM method. A null effect (beta = 0) suggests no association between the exposure and outcome, while positive (beta > 0) or negative (beta < 0) values indicate a risk factor or protective effect, respectively. Sensitivity analyses were conducted using the MR-Egger-intercept test and the leave-one-out method. The former assessed pleiotropy, while the latter evaluated the heterogeneity among genetic variants using Cochran Q statistic.^[21]^ The leave-one-out analysis was used to confirm that the results were not driven by any individual SNP.

### 2.6. Survival analysis

To assess the association with patient survival, the target gene was analyzed with gene expression profiling interactive analysis (GEPIA), a commonly employed web-based tool for cancer genomics (http://gepia2.cancer-pku.cn/#survival).

### 2.7. Ethical approval

The data used in this study were all from public databases, therefore ethical approval is not required.

## 3. Results

### 3.1. Analysis of GBD data

Analysis of the GBD data revealed that the number of global EC-related deaths increased from 356,263 (95% CI, 319,363–390,154) in 1990 to 538,602 (95% CI, 475,944–603,406) in 2021, with the average annual decrease in the EAPC for deaths being 0.31%. Additionally, the number of DALY cases increased from 9,753,566 (95% CI, 8,719,319–10,739,561) in 1990 to 12,999,256 (95% CI, 11,522,861–14,605,268) in 2021, with the average annual decrease in the EAPC for DALYs being 0.37% (Table 1).

**Table 1:** GBD data analysis on EC.

Deaths			Percentage change in ASRs from 1990–2021	DALYs		Percentage change in ASRs from 1990–2021
	Number of 1990	Number of 2021		Number of 1990	Number of 2021	
Global	356,263 (319,363–390,154)	538,602 (475,944–603,406)	−0.31 (−041 to −0.20)	9,753,566 (8719319–10739561)	12,999,265 (11522861–14605268)	−0.37 (−0.47 to −0.26)
Male	247,361 (220,766–278,717)	399,796 (343,473–459,871)	−0.25 (−0.39 to −0.10)	7,034,952 (6231588–7954187)	9,889,701 (8502607–11434422)	−0.33 (−0.46 to −0.18)
Female	108,901 (75497–125311)	138,806 (107414–161288)	−0.42 (−0.51 to −0.29)	2,718,614 (1845361–3147281)	3,109,564 (2478471–3575202)	−0.46 (−0.55 to −0.32)

ASRs = age-standardized rates¸ DALYs = disability-adjusted life years, EC = esophageal cancer, GBD = global burden of disease.

**Figure 2.**
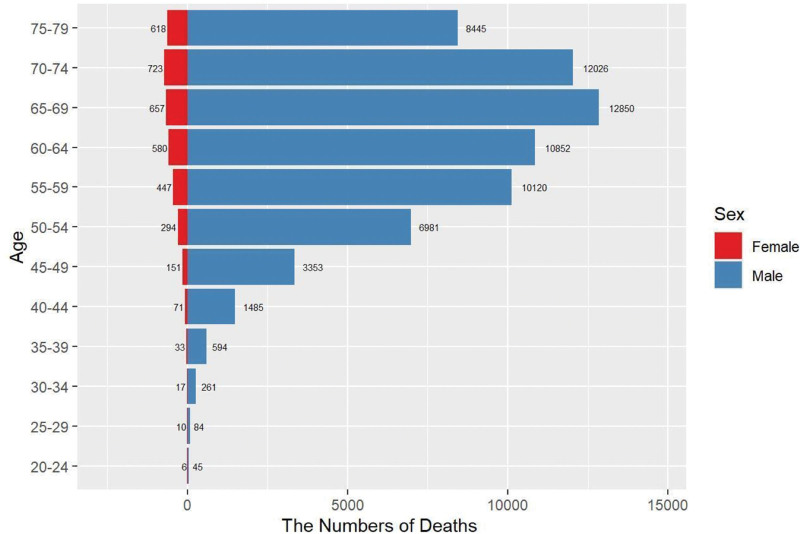
Age and sex distribution of global deaths number of EC. Trends in the number of deaths for EC across different genders (female and male) by age groups from 15 to 85 yr. EC = esophageal cancer.

Further analysis in terms of gender revealed that the number of cases and age-standardized rates were higher in females than in males. However, the EAPCs for deaths and DALYs decreased gradually in both genders (Fig. 2).

**Figure 3.**
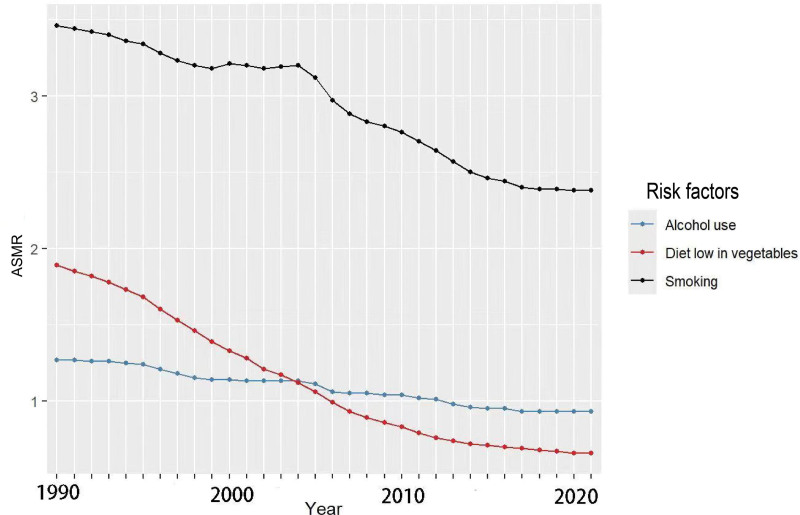
The trend of ASMR changes corresponding to the 3 risk factors of alcohol consumption, insufficient vegetable intake, and smoking from 1990 to 2020. ASMR = age-standardized mortality rate.

The identified risk factors included smoking, dietary habits, and alcohol consumption. The ASMRs for these risks gradually declined from 1990 to 2021, and fluctuated on a year-to-year basis, with smoking being the most significant contributor. The number of EC-related deaths attributed to smoking increased from 136,450 in 1990 to 205,463 in 2021, despite the fact that the mortality rate due to smoking was higher in 1990 (3.46) than in 2021 (2.38). A similar trend was observed for alcohol consumption, with the number of deaths increasing from 51,624 in 1990 to 81,227 in 2021, although the mortality rate was higher in 1990 (1.27) than in 2021 (0.93) (Fig. 3). The findings further revealed that the decline in the mortality rate attributed to dietary habits was more rapid compared to the decline in mortality rates attributed to smoking and alcohol consumption.

**Figure 4.**
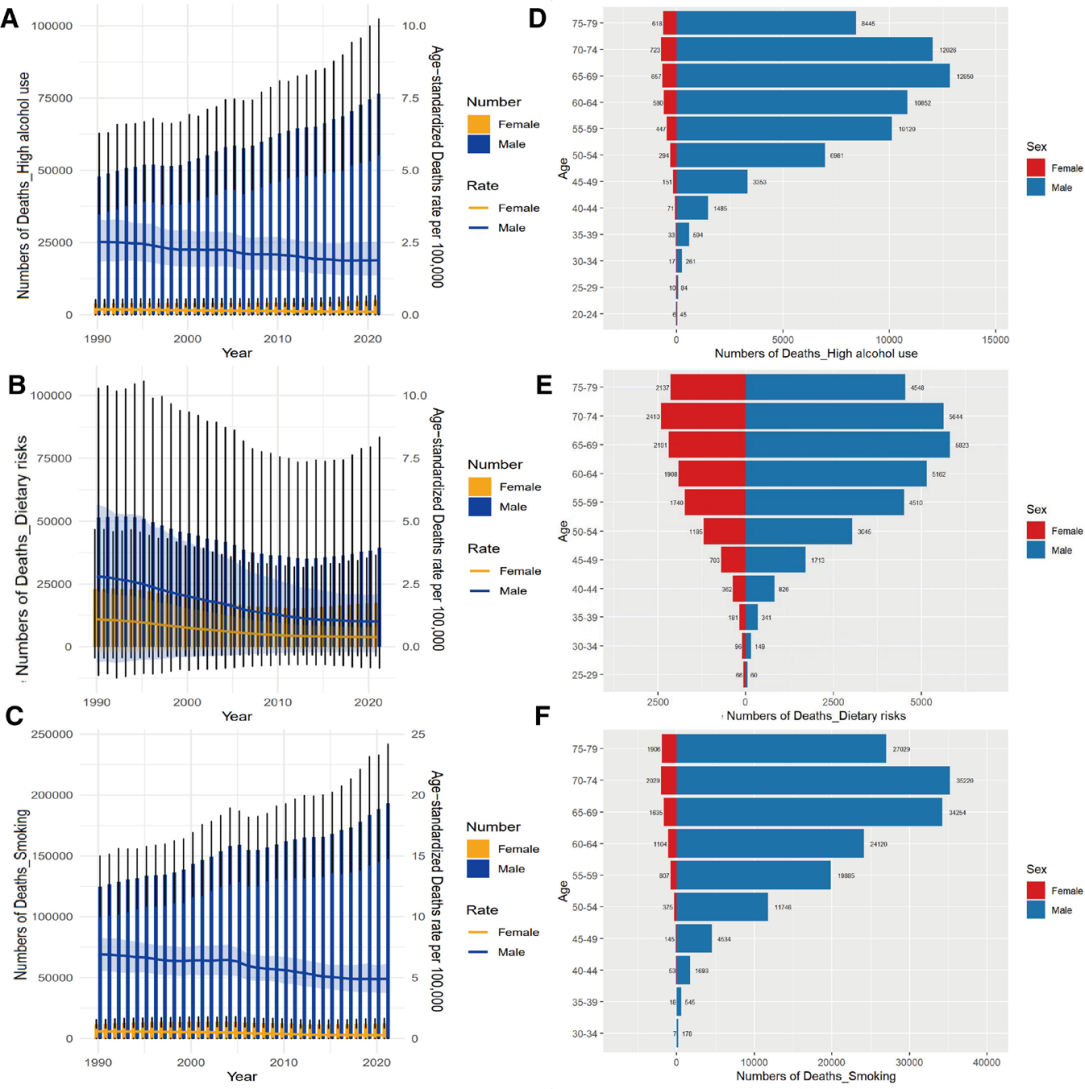
Biaxial graph for high alcohol use risks, dietary risks, and smoking risks and age, sex’s distribution of global deaths number of high alcohol use risks, dietary risks, and smoking risks. Trends in the number and age-standardized rates and their 95% UIs of death for high alcohol use risks (A), dietary risks (B), and smoking risks (C) across different genders (female and male) from 1990 to 2021, and trends in the number of deaths for high alcohol use risks (D), dietary risks (E), and smoking risks (F) across different genders (female and male) by age groups from 15 to 85 yr. UIs = uncertainty intervals

In 2021, the global incidence of EC-related deaths attributable to high alcohol consumption began to rise in individuals aged 20 to 24 years and peaked in the middle-aged group (65–69 years), and subsequently declined with advancing age. Similarly, the number of deaths attributable to dietary risks was highest in individuals aged 65 to 69 years, and subsequently declined in the higher age groups. Analysis of the global incidence of EC-related deaths attributable to smoking revealed that the number of deaths peaked in individuals aged 70 to 74 years (Fig. 4D–F). These 3 risk factors exhibited similar trends across different age groups, with the number of deaths in males being notably higher than that in females. Additionally, the male-to-female death ratio was smallest for dietary risk.

The number of EC-related deaths exhibited a significant decline over the years in both males and females. However, the decline in the mortality rate was less pronounced in women, potentially due to the lower baseline number of deaths in this population. Similarly, the difference in EC-related deaths between males and females attributable to dietary risk was smaller than that of the 2 other risk factors, which was likely due to the fact that men have a higher tendency to smoke and consume alcohol than women (Fig. 4A–C).

### 3.2. Univariate MR analysis

**Figure 5.**
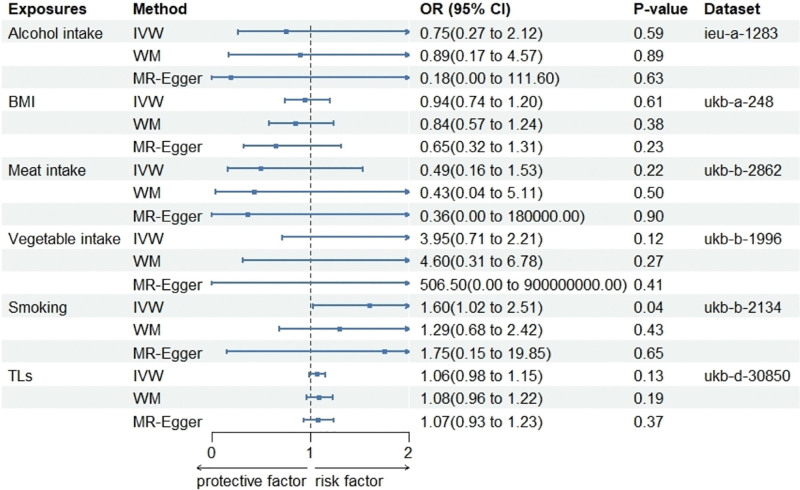
Forest plot of MR analyses results. The “Dataset” column signifies the dataset’s GWAS identifier in the IEU database or data sources. Results accompanied by confidence intervals exceeding “1” are deemed statistically nonsignificant. MR = Mendelian randomization.

Alcohol consumption, BMI, meat intake, consumption of vegetables, smoking, and TLs were selected as the exposure variables for the univariate MR analysis conducted in this study, and the value of the F-statistic associated with the analyzed SNPs was higher than 10. The results of MR analysis demonstrated that smoking exhibited a causal relationship with the risk of EC. Analysis with the IVW method revealed that the risk of EC due to smoking increased by 60% per increment in the standard deviation (OR = 1.60; 95% CI, 1.02–2.51; *P* = .04). The results of both the WM and MR-Egger regression methods were in agreement with those obtained from the IVW model. However, the other exposure factors selected herein did not exhibit statistically significant associations with the risk of EC (Fig. 5).

### 3.3. Sensitivity analyses

#### 3.3.1. MR-Egger intercept test and Q test

The heterogeneity among the genetic variants was assessed using Cochran *Q* test (Table S1–6, Supplemental Digital Content, https://links.lww.com/MD/R463). At the same time horizontal pleiotropy was evaluated using the MR-Egger regression method, which indicated the absence of directional pleiotropy. The detailed results are available in the supplementary section.

#### 3.3.2. Leave-one-out analysis

Leave-One-Out analysis of the MR data for each dataset revealed that the exclusion of individual SNP sequences did not alter the consistent risk estimates associated with EC in relation to alcohol consumption, smoking, BMI, dietary patterns, and TLs. The detailed results are available in the supplementary section.

#### 3.3.3. Meta-analysis of MR

**Figure 6.**
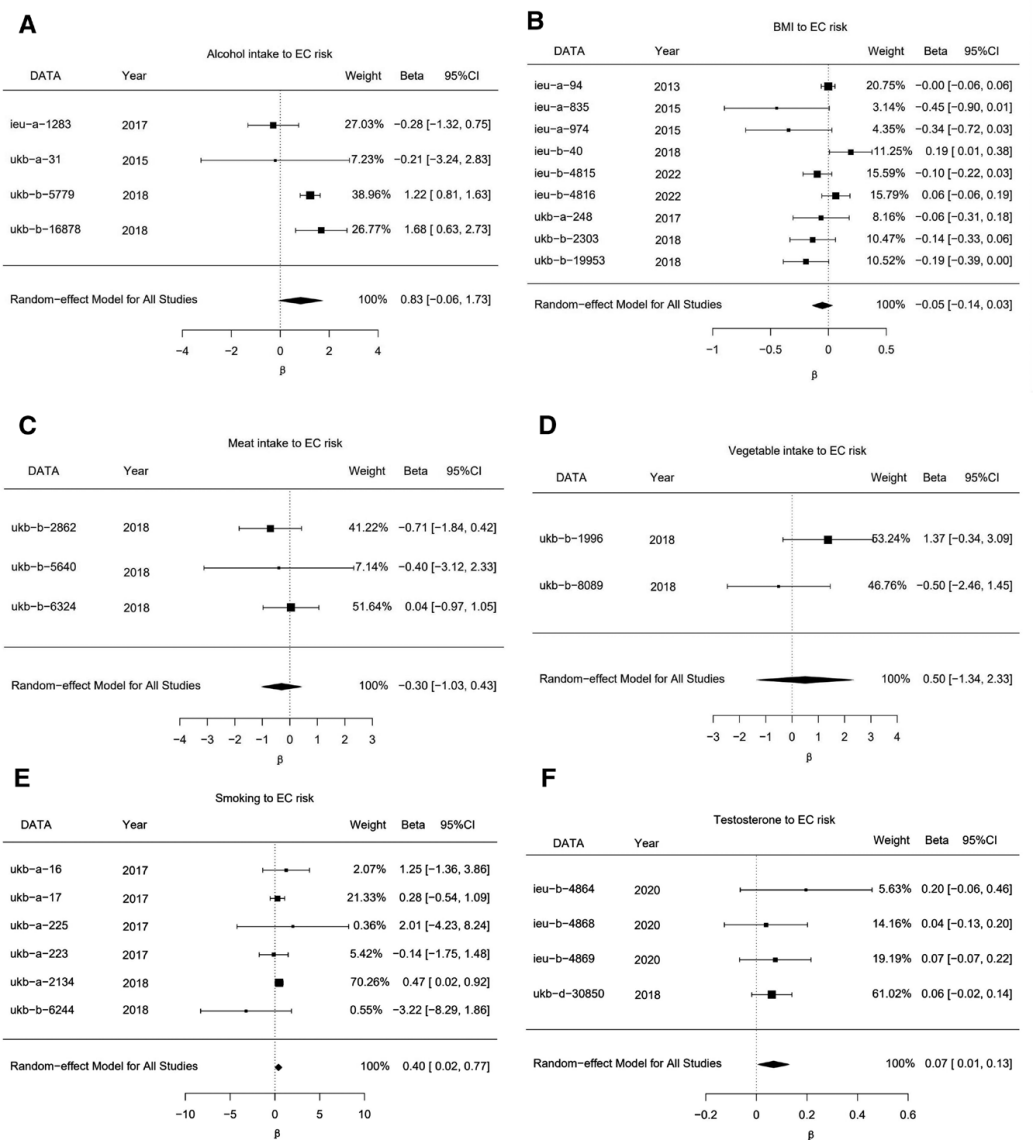
Meta analyses for exposures. (A) forest plot of Meta result for alcohol intake datasets; (B) forest plot of Meta result for BMI datasets; (C) forest plot of Meta result for meat intake datasets; (D) forest plot of Meta result for vegetable intake datasets; (E) forest plot of Meta result for smoking intake datasets; (F) forest plot of Meta result for TLs datasets. The “DATA” column indicates the dataset’s GWAS ID in the IEU database or data sources. BMI = body mass index, TLs = testosterone levels.

A meta-analysis incorporating the outcomes obtained by IVW analysis of multiple datasets was performed to substantiate our findings and mitigate any potential biases arising due to the implementation of a single dataset. The results of meta-analysis for alcohol intake, BMI, meat intake (including pork and beef), consumption of vegetables and smoking were consistent with the findings of univariate MR analysis. Meta-analysis of TLs revealed that although the individual datasets did not yield statistically significant findings, the results of pooled analysis demonstrated a modest yet statistically significant causal relationship between TLs and the risk of EC (Fig. 6). The results of meta-analysis indicated that the risk of EC exhibited a strong positive relationship with the TLs (*P* = .03; beta = 0.07; 95% CI, 0.006–0.13), and the frequency of smoking (*P* = .03; beta = 0.39; 95% CI, 0.02–0.78). However, the risk of EC was not positively associated with alcohol consumption (*P* = .07; beta = 0.83; 95% CI, −0.06–1.73) and the BMI (*P* = .23; beta = 0.05; 95% CI, −0.14–0.03). Additionally, the risk of EC did not have any significant causal relationship with the consumption of meat (*P* = .42; beta = -0.3; 95% CI, −1.02–0.43) or vegetables (*P* = .6; beta = 0.5; 95% CI, −1.34–2.33). The results of the analyses conducted on these datasets withstood sensitivity assessments, thereby confirming the robustness of the conclusions. The results of meta-analysis are presented in detail in the supplementary section (Table S7, Supplemental Digital Content, https://links.lww.com/MD/R463).

### 3.4. Survival analysis

**Figure 7.**
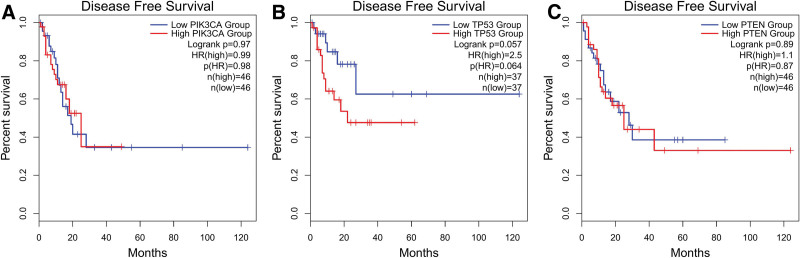
Survival analysis of PTEN, PIK3CA, and TP53, (A) survival analysis of PIK3CA protein, (B) survival analysis of TP53, (C) survival analysis of PTEN protein.

We performed a Survival Analysis of 3 proteins with established relationships to testosterone hormone. The results indicated that PTEN and PIK3CA proteins had no statistically significant impact on the survival of EC patients, while the TP53 protein exhibited a suggestive adverse effect on patient survival (Fig. 7).

## 4. Discussion

The GBD database was utilized in this study to identify potential risk factors for EC, which were subsequently subjected to MR analysis to determine their direct causal relationships with EC risk. A key objective of the study was to elucidate the potential factors contributing to the observed gender disparities in EC incidence. Analysis of the GBD data revealed statistically significant associations between EC risk and smoking, alcohol consumption, and poor dietary habits. Additionally, EC-related mortality was markedly higher in males than in females. MR analysis further demonstrated that smoking and elevated TLs had significant positive causal effects on EC risk. In contrast, no statistically significant causal relationships were found between EC and alcohol consumption, BMI, red meat intake, or vegetable consumption. These findings suggest that elevated TLs in males and smoking are the primary direct causal factors underlying the gender disparities in EC risk, while alcohol use, BMI, and dietary habits do not appear to have direct causal effects.

This study conducted a comprehensive investigation of risk factors associated with EC. While previous research utilizing GBD data has also identified significant gender disparities in EC risk,^[22]^ it has been suggested that unhealthy behaviors such as smoking and alcohol consumption may contribute to these differences.^[23]^ However, these earlier studies did not examine whether such lifestyle factors have a direct causal effect on EC risk. Furthermore, confounding variables – such as socioeconomic status – may influence the observed associations between unhealthy behaviors and EC risk, potentially leading to biased interpretations. To address these limitations, the present study employed MR analysis on risk factors identified from the GBD data to more robustly assess their direct causal effects on the development of EC.

The findings of this study are partially supported by existing literature. Our results regarding the causal effects of various risk factors – such as unhealthy behaviors (e.g., smoking and alcohol consumption) and dietary patterns (e.g., red meat and vegetable intake) – are consistent with those reported in several previous studies, including those by Yuan et al, Zou et al, and Yun et al.^[24–26]^ However, some prior studies, such as those by Liu et al and Lee et al, present findings that diverge from the results of our MR analysis.^[27,28]^ For example, Liu and Zhang^[27]^ conducted a meta-analysis of observational studies and concluded that there was no statistically significant association between TLs and EC risk. Similarly, Lee and Jiang,^[28]^ in an analysis involving approximately 1 million participants across 44 cohorts, identified a J-shaped association between BMI and EC risk. While these findings differ from ours, it is important to note that both studies relied on observational data and may be subject to residual confounding from factors such as socioeconomic status and dietary intake, which can compromise causal inference. In contrast, the MR approach employed in our study helps to minimize such biases, providing more robust evidence for causal relationships.

Notably, the MR analysis conducted by Chang et al investigated the association between TLs and pan-cancer risk. However, their study did not identify a causal relationship between TLs and the risk of EC,^[29]^ which differs from the findings of the present study. This discrepancy may be attributable to biases introduced by relying on a single dataset. It is plausible that inter-year variability, institutional differences, and population heterogeneity – such as ethnicity – within a single dataset could substantially affect MR outcomes and introduce confounding bias. In our analysis, when individual datasets were used, no significant causal association between TLs and EC risk was observed. However, when results from multiple datasets were combined, TLs demonstrated a significant causal relationship with EC risk. These findings suggest that integrating data from diverse sources enhances the robustness and credibility of MR conclusions.

Previous studies have consistently demonstrated a pronounced gender disparity in the risk of developing EC. The present study suggests that this difference may be attributed, at least in part, to a direct causal relationship between TLs and EC risk. Furthermore, it is hypothesized that the underlying mechanism may involve lipoprotein metabolism. Lee et al reported a significant association between TLs and lipoprotein profiles, characterized by a negative correlation with triglyceride deciles, a positive correlation with high-density lipoprotein cholesterol deciles, and an inverted U-shaped relationship with low-density lipoprotein deciles.^[30]^ Supporting this, Liu et al conducted a MR analysis and confirmed a direct causal relationship between TLs and specific lipoprotein levels.^[31]^ Additionally, earlier MR studies indicated that both triglycerides and low-density lipoprotein may serve as protective factors against EC. Collectively, these findings suggest that the interaction between testosterone and lipoproteins may play a role in modulating EC risk.^[32]^ Thus, the hypotheses proposed in this study regarding the influence of testosterone and the potential mechanistic pathways involved in EC development appear to be biologically plausible.

In light of our survival analysis of 3 proteins, PTEN, PIK3CA, and TP53[8–10], which exhibit a definitive correlation with TLs, we propose that TP53 may act as a mediator in the causal relationship between TLs and the risk of EC. This implication may be attributed to the pivotal role of the TP53 protein in the p53 signaling pathway. Consequently, this investigation postulates that the causal influence of TLs on EC may be intimately linked to the p53 signaling pathway. We urge future researchers to undertake more comprehensive explorations in this domain.

In the present study, potential risk factors for EC were first identified using data from the GBD database, followed by a secondary analysis using MR. This methodological approach helps to minimize the influence of confounding variables on the observed associations between exposures and outcomes, thereby enhancing the rigor and reliability of causal inference. To further strengthen the analysis, datasets from diverse institutions, time periods, and ethnic groups were incorporated. A meta-analysis was then conducted to synthesize the results across these datasets, ensuring a comprehensive and robust evaluation of the MR findings. This integrative approach effectively reduces the potential bias associated with relying on a single dataset, thereby increasing the overall credibility and generalizability of the study’s conclusions.

However, this study has several limitations that warrant consideration. First, the complex biological effects of smoking and TLs present significant challenges in elucidating the precise causal mechanisms linking these factors to the risk of EC. While the study offers plausible hypotheses regarding these mechanisms, they remain speculative and have yet to be substantiated by experimental or mechanistic studies. Second, TLs were used as a proxy for hormonal differences between sexes to investigate gender disparities in EC risk. However, hormonal differences between males and females are multifaceted, and relying solely on TLs may oversimplify these distinctions, potentially limiting the robustness of the conclusions. Furthermore, gender differences in EC risk are not solely attributable to hormonal variation, but may also be influenced by a range of factors such as lifestyle, physical activity, and environmental exposures. To address these limitations, future studies should adopt a multidisciplinary approach that integrates bioinformatics with experimental validation to achieve a more comprehensive understanding of the roles of TLs and smoking in EC pathogenesis. Furthermore, this study omitted the analysis of EC precursors, such as Barrett esophagus, gastroesophageal reflux disease, and corrosive ulcers, and did not account for the coexistence of malignant tumors, such as those in the stomach, which may introduce inaccuracies in predicting risk factors for EC. We recommend that future research undertake a more exhaustive analysis building upon this study. Additionally, constrained by the limitations of GWAS data, this study excluded clinical information, such as treatment modalities, leading to a deficiency in clinical significance. We intend to integrate clinical data analysis with subsequent research to augment the clinical relevance of the findings.

In conclusion, further research is needed to gain deeper insights into the marked gender disparities in EC risk and to generate actionable clinical insights and public health policy recommendations, thereby advancing health strategies and deepening our understanding of related diseases.

## 5. Conclusions

Significant gender-based disparities exist in EC incidence, with males exhibiting markedly higher risk. This divergence could be attributed principally to direct causal effects of 2 factors: smoking and biologically inherent male TLs. Tobacco use demonstrates a strong male-predominant exposure pattern and directly contributes to EC development. Concurrently, characteristically elevated male TLs exhibit direct causal effects on EC pathogenesis, independent of other variables.

Regarding other exposures, factors such as alcohol consumption, BMI, and dietary habits do not exert significant effects on EC risk disparities. Population studies confirm alcohol consumption lacks differential gender-based impact sufficient to explain EC disparity. Similarly, BMI variations show comparable EC risk associations across genders. Dietary habits analyses reveal no statistically significant gender-interaction effects for major food groups or patterns. Multivariate adjustments for smoking and TLs further nullify these factors’ marginal contributions.

Thus, the EC gender gap stems significantly from smoking prevalence and physiological TL differences in males, not from alcohol intake, body mass variations, or nutritional patterns.

Supplemental digital contents “Supplement 1 & 2” are available for this article (https://links.lww.com/MD/R461; https://links.lww.com/MD/R462).

## Acknowledgments

We appreciate the linguistic assistance provided by TopEdit (www.topeditsci.com).

## Author contributions

**Data curation:** Jiale Cui.

**Formal analysis:** Xiaomeng Zhang.

**Funding acquisition:** Lei Li.

**Resources:** Lei Li.

**Software:** Jiale Cui.

**Supervision:** Jiale Cui, Rong Zhang.

**Validation:** Rong Zhang.

**Visualization:** Rong Zhang.

**Writing – original draft:** Jiale Cui, Xiaomeng Zhang, Ziyi Zhang, Meixuan Li, Rui He, Peirui Jiang.

**Writing – review & editing:** Jiale Cui, Ziyi Zhang, Lei Li.

## Supplementary Material






